# Combined Theoretical and Experimental Study of the Moiré Dislocation Network at the SrTiO_3_-(La,Sr)(Al,Ta)O_3_ Interface

**DOI:** 10.1021/acsami.3c10958

**Published:** 2023-11-09

**Authors:** Chiara Ricca, Elizabeth Skoropata, Marta D. Rossell, Rolf Erni, Urs Staub, Ulrich Aschauer

**Affiliations:** † Department of Chemistry and Biochemistry, University of Bern, Freiestrasse 3, CH-3012 Bern, Switzerland; ‡ Swiss Light Source, Paul Scherrer Institut, Forschungsstrasse 111, 5232 Villigen PSI, Switzerland; § Electron Microscopy Center, Empa, Swiss Federal Laboratories for Materials Science and Technology, Überlandstrasse 129, 8600 Dübendorf, Switzerland; ∥ Department of Chemistry and Physics of Materials, University of Salzburg, Jakob-Haringer-Street 2A, A-5020 Salzburg, Austria

**Keywords:** oxide interface, Moiré lattice, interfacial dislocations, chemical composition, X-ray diffraction, electron microscopy, molecular dynamics, density functional theory

## Abstract

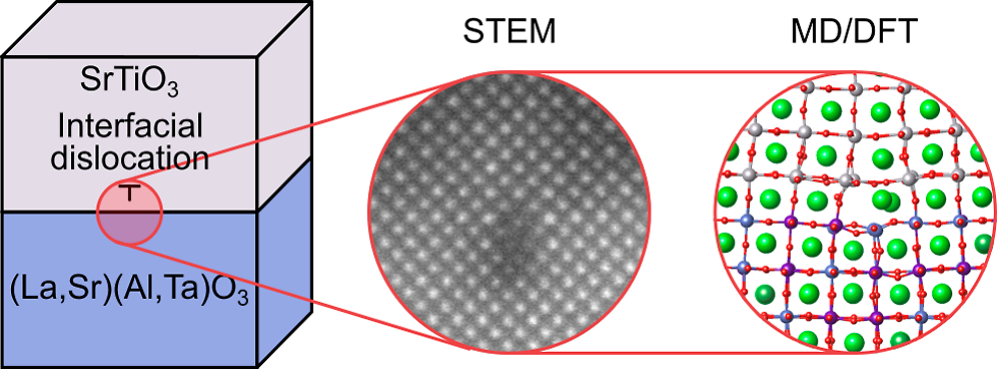

Recently, a highly ordered Moiré dislocation lattice was identified at the interface between a SrTiO_3_ (STO) thin film and the (LaAlO_3_)_0.3_(Sr_2_TaAlO_6_)_0.7_ (LSAT) substrate. A fundamental understanding of the local ionic and electronic structures around the dislocation cores is crucial to further engineer the properties of these complex multifunctional heterostructures. Here, we combine experimental characterization via analytical scanning transmission electron microscopy with results of molecular dynamics and density functional theory calculations to gain insights into the structure and defect chemistry of these dislocation arrays. Our results show that these dislocations lead to undercoordinated Ta/Al cations at the dislocation core, where oxygen vacancies can easily be formed, further facilitated by the presence of cation vacancies. The reduced Ti^3+^ observed experimentally at the dislocations by electron energy-loss spectroscopy is a consequence of both the structure of the dislocation itself and of the electron doping due to oxygen vacancy formation. Finally, the experimentally observed Ti diffusion into the LSAT around the dislocation core occurs only together with cation vacancy formation in the LSAT or Ta diffusion into STO.

## 1 Introduction

Complex transition metal perovskite oxides are a versatile class of materials with a wide spectrum of functional properties. They can be insulating, semiconducting, or metallic and show technologically relevant phenomena such as magnetism, ferroelectricity, or the more exotic high-temperature superconductivity and colossal magnetoresistance.^1−4^ These properties are the result of a complex interplay of charge, orbital, spin, and lattice degrees of freedom^1^ and depend strongly on strain and the defect chemistry.^5−7^ The structural compatibility between different perovskites allows them to be stacked on top of each other, and the advances in deposition techniques enabled the fabrication of complex multifunctional heterostructures with relative ease. These heterostructures often give rise to interesting, novel, and unexpected physical phenomena emerging at the interface where materials with different structural and electronic properties meet: quasi-two-dimensional (2D) electron gas, colossal ionic conductivity, giant thermoelectric effect, or resistance switching.^1,3,8−12^

Recently, a highly ordered Moiré lattice has been identified at the interface between SrTiO_3_ (STO) and (LaAlO_3_)_0.3_(Sr_2_TaAlO_6_)_0.7_ (LSAT) by high-resolution X-ray diffraction reciprocal space mapping.^13^ A 30 nm thick film of STO was grown on LSAT (001) by pulsed laser deposition, followed by 12 h of annealing at 1200 °C and ambient pressure. STO (*a*_STO_ = 3.905 Å) and LSAT (*a*_LSAT_ = 3.869 Å) both have a cubic lattice with a small mismatch of 0.93%. The high-temperature annealing allows the almost complete relaxation of the STO film, which results in the appearance of the Moiré pattern. This newly formed 2D pattern has a Moiré lattice constant of approximately 40 nm, corresponding to 106/107 unit cells of STO/LSAT, necessary to compensate for the small lattice mismatch between the two materials. Scanning transmission electron microscopy (STEM) images suggest that this periodicity is related to the appearance of a network of edge dislocations at the interface that has the same periodicity as the Moiré pattern.

The ability to form such ordered superlattices with a 2D network of line defects at complex perovskite oxide interfaces could be an emerging avenue to induce new interfacial functionalities with unforeseen potential applications, such as 2D ferroics, 2D grid conductivity along the defect lines, or ferroelectric three-dimensional (3D) vortex structures. These interfacial phenomena are a consequence of spin and charge interactions at the interface, which are in turn controlled by the local atomic arrangement. Hence, a detailed understanding of the atomic structure and electronic properties of the STO/LSAT interface in the presence of dislocations is key to interpreting the behavior of this heterostructure and to enhance its functional properties.

Given that the oxide defect chemistry and diffusion are known to be heavily affected by strain^14−17^ and since dislocations are surrounded by strong strain fields, one would expect preferential defect formation and segregation around dislocations. Indeed, recent computational studies have focused not only on describing the structure of interfacial misfit dislocations^18,19^ but also on their interaction with dopants, defects, and defect clusters.^20−22^ These classical potential-based calculations, however, always treat charged point defects since whole ions are removed. Electronic structure density functional theory (DFT) calculations would be necessary to account for the formation of neutral or not fully ionic defects. In the present case, DFT is, however, limited by the large size of the supercells required to describe the periodicity of the Moiré lattice and misfit dislocations. In addition, the huge configurational space of point defects and point-defect clusters in the symmetry-broken environment represents a challenge, given the computational cost of DFT.

To overcome these challenges, we use classical molecular dynamics (MD) to obtain a dislocation structure with the correct long-range strain field of the Moiré interface with misfit dislocations and combine it with DFT calculations on cluster models extracted from these classically relaxed structures. The space of possible defects and their location around the dislocation are reduced by experimental information obtained from chemical mapping by electron energy-loss (EELS) and energy-dispersive X-ray (EDX) spectroscopies. The results lead to a detailed understanding of the atomic-scale defect structure and the resulting electronic properties around the interfacial dislocation network that will guide the design and optimization of these promising heterostructures.

## 2 Methods

### 2.1 Computational Details

#### 2.1.1 Dislocation Models

**Figure 1:**
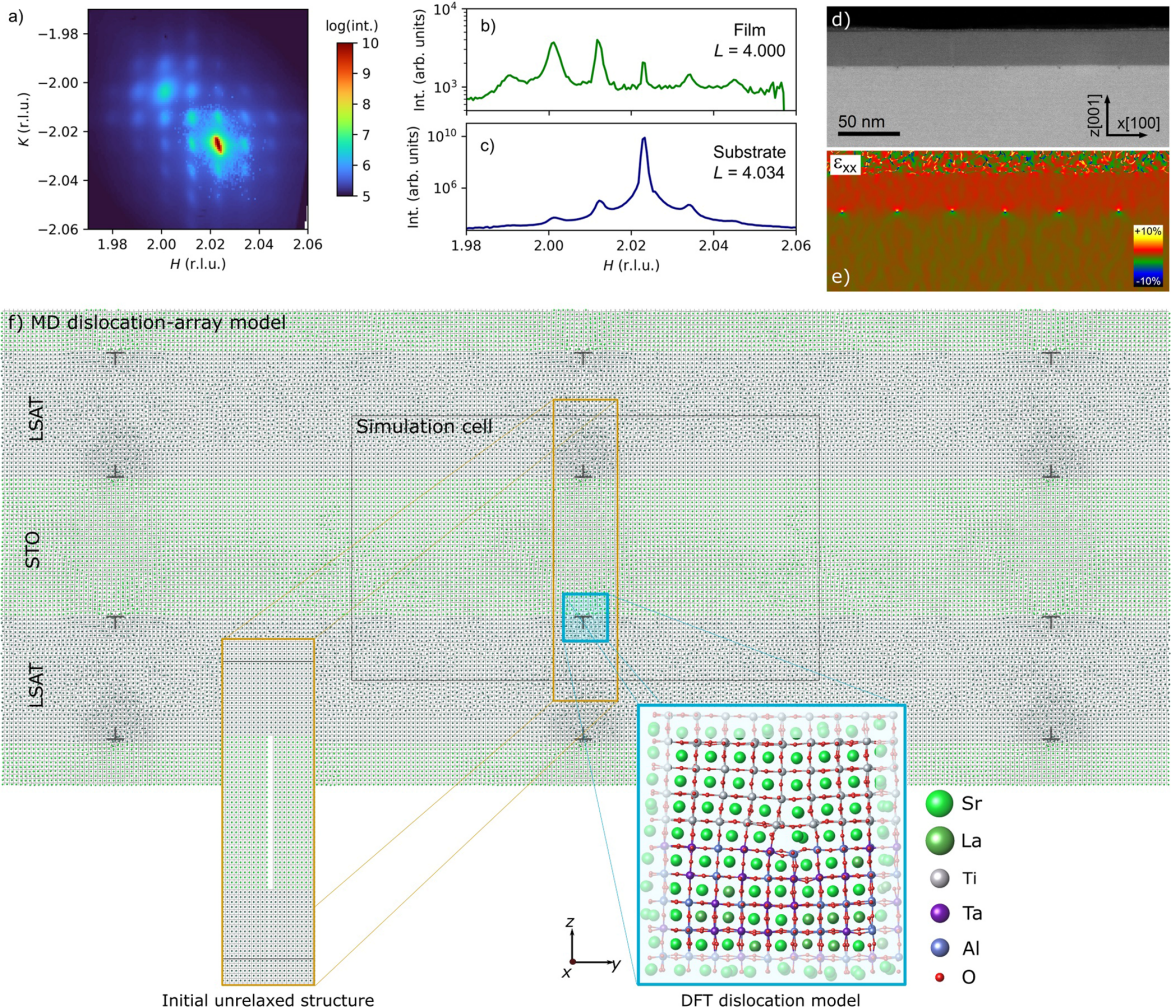
(a) In-plane projection of the X-ray diffraction of the (22̅4) reflection of SrTiO_3_/LSAT thin films with a Moiré lattice. Line cuts through the (b) film (*L* = 4.000) and (c) substrate (*L* = 4.034) reflections show the periodicity of the Moiré lattice. (d) HAADF-STEM image of a STO thin film grown on a LSAT (001) substrate and (e) corresponding strain map along the *x* direction calculated by geometric phase analysis. The misfit dislocations appear as butterflylike features at the STO/LSAT interface with a compression region (in blue) and a tensile region (in yellow). (f) Structure of the periodically repeated relaxed MD model of the dislocation array at the SrTiO_3_/LSAT interface with the dislocations and the (2 × 107 × 60) simulation cell highlighted. Only the B–O bonding network and the A-site atoms are shown for clarity. Insets show the initial unrelaxed part of the structure with the removed STO plane and the extracted DFT cluster model, respectively. In the DFT model, the faded-out atoms are fixed during DFT geometry optimization to impose elastic boundary conditions.

The simulation of these dislocation arrays is complicated by their nonperiodic nature along the interface normal and by their large ordering period along the interface. An appropriate description along both of these directions is, however, crucial to accurately determine the long-range strain field associated with the dislocation. The interfacial Moiré periodicity can be determined by X-ray diffraction experiments (see Figure 1a–c for a plane-view and line cuts through the reciprocal space volume) and best fits simulated diffraction patterns for a 106/107 type Moiré motif.^13^ This periodicity with a Moiré lattice constant of 40.8 nm also compensates for the 0.93% lattice mismatch between the STO film and the LSAT substrate. The associated interfacial dislocation structure matches this periodicity as shown by the high-angle annular dark-field (HAADF)-STEM image and associated strain map in Figure 1d,e.

In order to obtain a reliable computational description of the 2D Moiré pattern at the interface of a thin film of STO on LSAT (001), we employed the simulation setup schematically shown in Figure 1f, which is a combination of the simulation approaches suggested in refs (20), (23), and (24). We started by creating two dislocations with opposite Burgers vectors in a simulation box with dimensions ∼0.75 nm × 41.50 nm × 23.51 nm (approximately 64,000 atoms) corresponding to a 2 × 107 × 60 supercell of a 5-atom cubic perovskite unit cell. The number of cells along the *y* axis allows us to reproduce the observed periodicity of the Moiré superlattice, while along the *z* axis, we stacked 30 STO on 30 LSAT layers. This means that the two dislocation cores are separated by about 11.76 nm along the *z* axis, which is sufficient to significantly suppress interactions between them. A missing plane is then created in the middle of the cell by removing one SrO and one TiO_2_ plane (see Figure 1f). The system is relaxed via classical MD (see below for details), during which the missing plane heals, forming two dislocations with opposite Burgers vectors at the upper and lower interfaces.

DFT calculations (see below for details) are then performed on a cluster model created from the final MD structure by extracting a section of 2 × 10 × 10 STO/LSAT unit cells around the bottom dislocation core (Figure 1f). This cell contains 950 atoms and 5 STO and 5 LSAT layers along the *z* axis. For the LSAT substrate, 18 La and 82 Sr, as well as 59 Al and 41 Ta atoms, were randomly introduced at the A and B sites, respectively, to obtain a charge-neutral LSAT layer with a composition (La_0.2_Sr_0.8_Al_0.4_Ta_0.6_O_6_) similar to that of the experiment (La_0.3_Sr_0.7_Al_0.3_Ta_0.7_O_6_). The cluster was periodically repeated along the dislocation line only (*x* axis), while in the perpendicular directions, only the periodicity of the electrostatic potential was imposed. Furthermore, atoms in the boundary region were kept fixed throughout the simulation to impose the elastic boundary conditions of the interface and dislocation environment. Within this approach, it is possible to avoid artifacts due to the interaction of the dislocation with its images in neighboring cells, thus isolating the dislocation core. The dislocation core is embedded in the correct long-range elastic field, allowing, at the same time, to minimize any spurious effect due to long-range electrostatic fields.^23^

Neutral vacancies (V_*X*_) were created by removing one *X* (*X* = O, La, Al, and Ta) atom from the DFT dislocation model. Ti-substitution at Al (Ti_Al_) or Ta (Ti_Ta_) sites and Ta-substitution of Ti atoms (Ta_Ti_) were also taken into account. Different possible vacancies and substitutional defect configurations involving different sites around the dislocation were considered. All data are available on the Materials Cloud Archive.^25^

#### 2.1.2 Molecular Dynamics

The LAMMPS^26^ code was used to perform the classical MD simulations. Interatomic interactions were described using a nonpolarizable rigid-ion model consisting of long-range electrostatic interactions between the nuclei and a short-range Buckingham potential with a cutoff radius of 12 Å
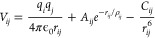
1where *q*_*i*/*j*_ is the atomic charge, *r*_*ij*_ is their separation, and *A*_*ij*_, ρ_*ij*_*,* and *C*_*ij*_ are the parameters of the short-range potential as reported in Table 1. The short-range parameters were derived by starting from the Lewis and Catlow^27^ set and using the GULP code^28−30^ to fit lattice parameters and elastic constants of STO and LSAT derived from DFT calculations (see below). For LSAT, we chose to describe the interaction between the O and A/B dummy atoms representing the average properties of the La/Sr and Al/Ta atoms occupying the A and B sites, respectively. MD simulations were performed in the canonical (NVT) ensemble with a Nose–Hoover thermostat and barostat. The system was allowed to relax first at 50 K for 50 ps and then at 1473 K for 130 ps before the temperature was reduced again to 50 K over 120 ps. In order to drain the kinetic energy released during closing the missing plane in a controlled way, a viscous damping force with a coefficient γ = 1.0 eV·ps/Å^2^ is applied to all atoms.

**Table 1:** Parameters of the Coulomb–Buckingham Potentials for STO and LSAT

*i*	*j*	*q*_*i*_ (e)	*A*_*ij*_ (eV)	ρ_*ij*_ (Å)	*C*_*ij*_ (eV/Å^–^^6^)
Sr	O	2.00	1324.77	0.3008	0.00
Ti	O	4.00	762.26	0.4014	0.00
A	O	2.18	2018.14	0.2876	0.00
B	O	3.82	867.03	0.3828	0.00
O	O	–2.00	22764.30	0.1490	31.15

DFT calculations to determine lattice parameters and elastic constants used in potential fitting were performed with the VASP code.^31−34^ We used the PBE^35^ exchange-correction functional together with PAW^36,37^ potentials with La(5s, 5d, 5p, and 6s), Sr(4s, 4p, and 5s), Al(3s and 3p), Ta(5p, 5d, and 6s), and O(2s and 2p) valence electrons and a plane-wave cutoff of 550 eV. For LSAT, we used a  supercell of the 5-atom cubic unit cell with composition La_3_Sr_13_Al_9_Ta_7_O_48_, the reciprocal space of which was sampled with a 4 × 4 × 4 Monkhorst–Pack^38^ mesh, while a 8 × 8 × 8 mesh was used for the 5-atom STO unit cell (3.90 Å × 3.90 Å × 3.90 Å). Structures were relaxed until forces converged below 10^–4^ eV/Å before elastic constants were determined using a central finite-differences approach with a step size of 0.015 Å.

#### 2.1.3 DFT Calculations

The DFT calculations on the cluster model were performed with the CP2K program package^39^ using the PBE^35^ exchange–correlation functional. The norm-conserving Goedecker–Teter–Hutter (GTH) pseudopotentials^40^ together with the GTH double-ζ polarized molecularly optimized basis sets^41^ and an energy cutoff of 750 Ry were applied. The convergence criterion for the self-consistent field method was set to 1 × 10^–6^ Ha, while atomic positions were relaxed within a force threshold of 10^–3^ eV/Å.

The defect formation energy (*E*_f_) of a neutral defect was calculated as described in ref (42)
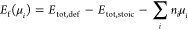
2where *E*_tot,def_ and *E*_tot,stoic_ are the DFT total energies of the defective system and of the stoichiometric cell, respectively. *n*_*i*_ indicates the number of atoms of a certain species *i* that is added (*n*_*i*_ > 0) or removed (*n*_*i*_ < 0) from the supercell to form the defect, while μ_*i*_ is the species’ chemical potential. For simplicity, we used the atomic energy of the corresponding reference phase for each element (metal La, Ta, Ti, Al, and molecular O_2_) as the chemical potential.

### 2.2 Experimental Methods

#### 2.2.1 X-ray Diffraction

X-ray diffraction was measured at the surface diffraction endstation of the materials science beamline at the Swiss Light Source. X-rays of 12.65 keV were focused with a beam size of 500 × 500 μm on the sample at room temperature. The (22̅4) reflection was measured with an area detector after alignment of the UB matrix with 3 orthogonal and 2 nonorthogonal reflections. The detector images were converted to reciprocal space according to Schlepütz et al.,^43^ and the data were analyzed using the xrayutilities package.^44^

#### 2.2.2 Scanning Transmission Electron Microscopy

Electron-transparent samples for STEM investigations were produced in a cross-section geometry using an FEI Helios 660 G3 UC dual-beam focused (Ga) ion beam instrument operated at 30 and 5 kV, after the deposition of C and Pt protective layers. HAADF STEM, EELS, and EDX were carried out using a probe aberration-corrected FEI Titan Themis microscope operated at 300 kV and equipped with a SuperX EDX system and a CEFID energy filter in combination with an ELA direct electron detector (for details, see ref (45)). For the HAADF-STEM data acquisition, a probe convergence semiangle of 26 mrad was set, and the inner angle of the annular semidetection range was 171 mrad. The EELS data were obtained with a collection semiangle of 35 mrad yielding an effective collection angle of about 29.5 mrad considering the energy range of the spectra.

A quantitative analysis of the lattice distortions at the dislocation cores was performed by means of peak-pair analysis (PPA), fitting the peaks corresponding to the atomic columns in the high-resolution HAADF-STEM images as described in ref (46). In particular, we analyzed the structural distortions by measuring the distance between the peaks corresponding to the atomic columns of the A sublattice.

## 3 Results and Discussion

### 3.1 Structure and Strain Field at the Dislocation Core

**Figure 2:**
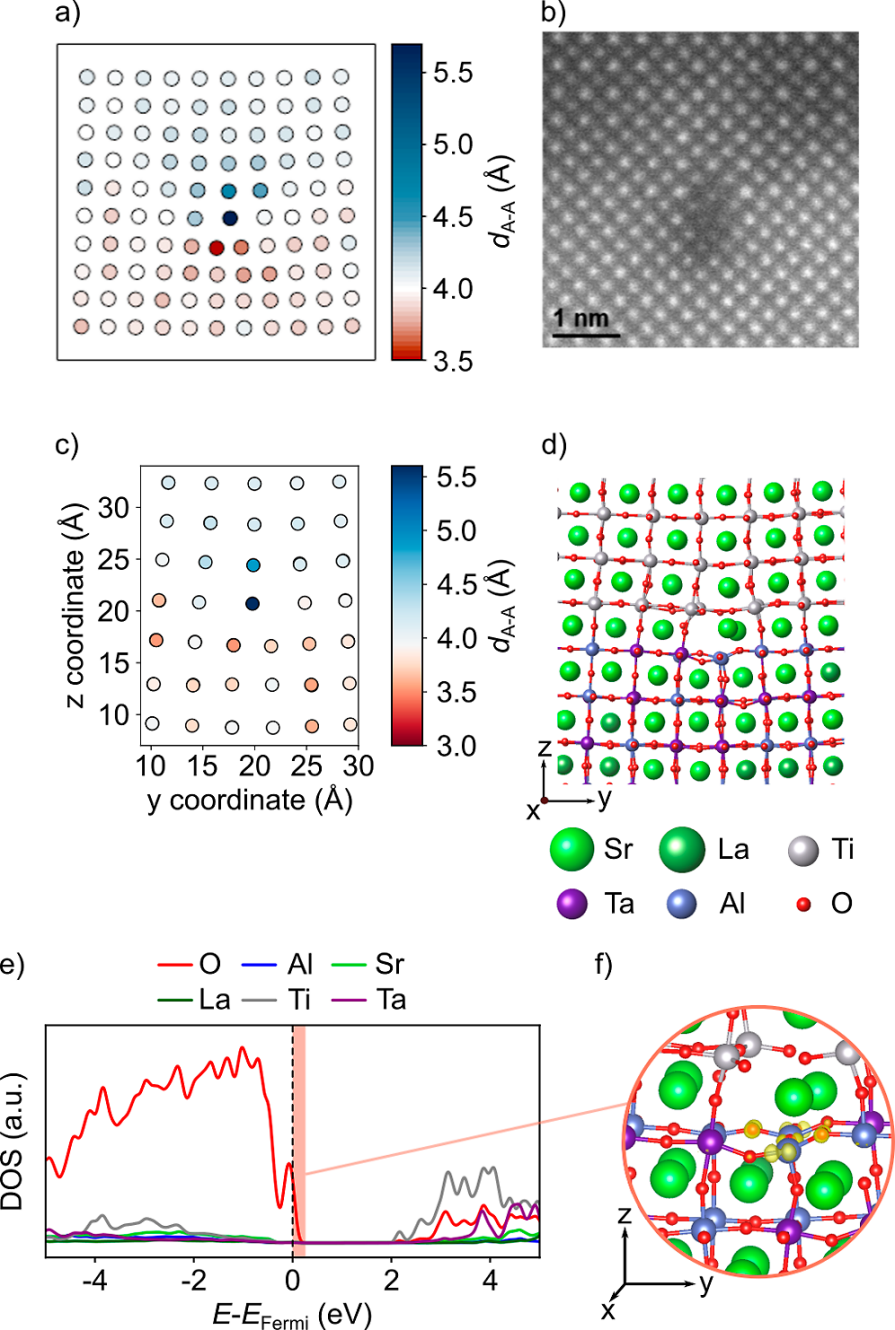
(a) Map of the interatomic distances (in Å) for the A sublattice along the horizontal direction as obtained by PPA from the HAADF-STEM image in (b). (c) Map of horizontal interatomic A–A distances extracted from the DFT-relaxed dislocation core structure shown in (d). Circles are located at the midpoint of A–A pairs and are color-coded according to the distance between that pair. (e) Electronic density of states (DOS) projected on the atoms at the dislocation core for the stoichiometric dislocation model. The vertical dashed line indicates the position of the Fermi level. (f) Charge density isosurface (10^–2^ e/Å^3^) in the energy range highlighted in red in (e).

As already discussed in ref (13), high-resolution STEM images of the annealed STO/LSAT samples reveal the presence of a highly ordered arrangement of edge dislocations with periodicity equivalent to the one of the Moiré lattice observed by high-resolution X-ray diffraction reciprocal space mapping. These dislocations form where the local structural mismatch between the two materials is the largest (see Figure 2b). In the present work, the HAADF-STEM images have been used to map the strain field around the dislocation core, as reported in Figure 2a. The strain field is, indeed, highly localized around the dislocation core, where two different strain regions can be identified: a tensile strain region in the STO film and a compressive strain region in the LSAT layer.

Figure 2d shows the final structure of the dislocation core obtained after DFT geometry optimization starting from the structure extracted from the MD simulations. The strain field associated with this model was computed by extracting A–A distances parallel to the interface, as shown in Figure 2c. In this case, the largest strains are also observed at the dislocation core with an average tensile/compressive strain in the STO/LSAT layer, respectively. Overall, the strain-field maps derived from experiments and DFT calculations show a qualitative agreement, allowing us to confidently use the DFT model to understand the structural properties of the dislocation core at the atomic level.

As shown in Figure 2d, highly strained Ti–O–Ti bonds are established across the missing planes, leaving the B cations of the extra Ta/AlO_2_ plane undercoordinated at the dislocation core. The missing Al–O bonds at the STO/LSAT interface induce hole doping of the system, as can be seen from the DOS projected on the atoms at the dislocation core (Figure 2e), where the Fermi level crosses the top of the valence band, which is formed by the O-2p states of the O atoms bonded to the undercoordinated B cations at the dislocation core (Figure 2f).

Despite the overall qualitative agreement, the dislocation core imaged by STEM shows contrast differences larger than expected from the structural and electronic changes in the DFT model, which could result from the DFT model being constructed with stoichiometric STO and LSAT, while point defects, such as oxygen and cation vacancies or substitutional defects, may be present in experiment.

### 3.2 Defect Chemistry of the Dislocation Core

#### 3.2.1 Oxygen Vacancies

**Figure 3:**
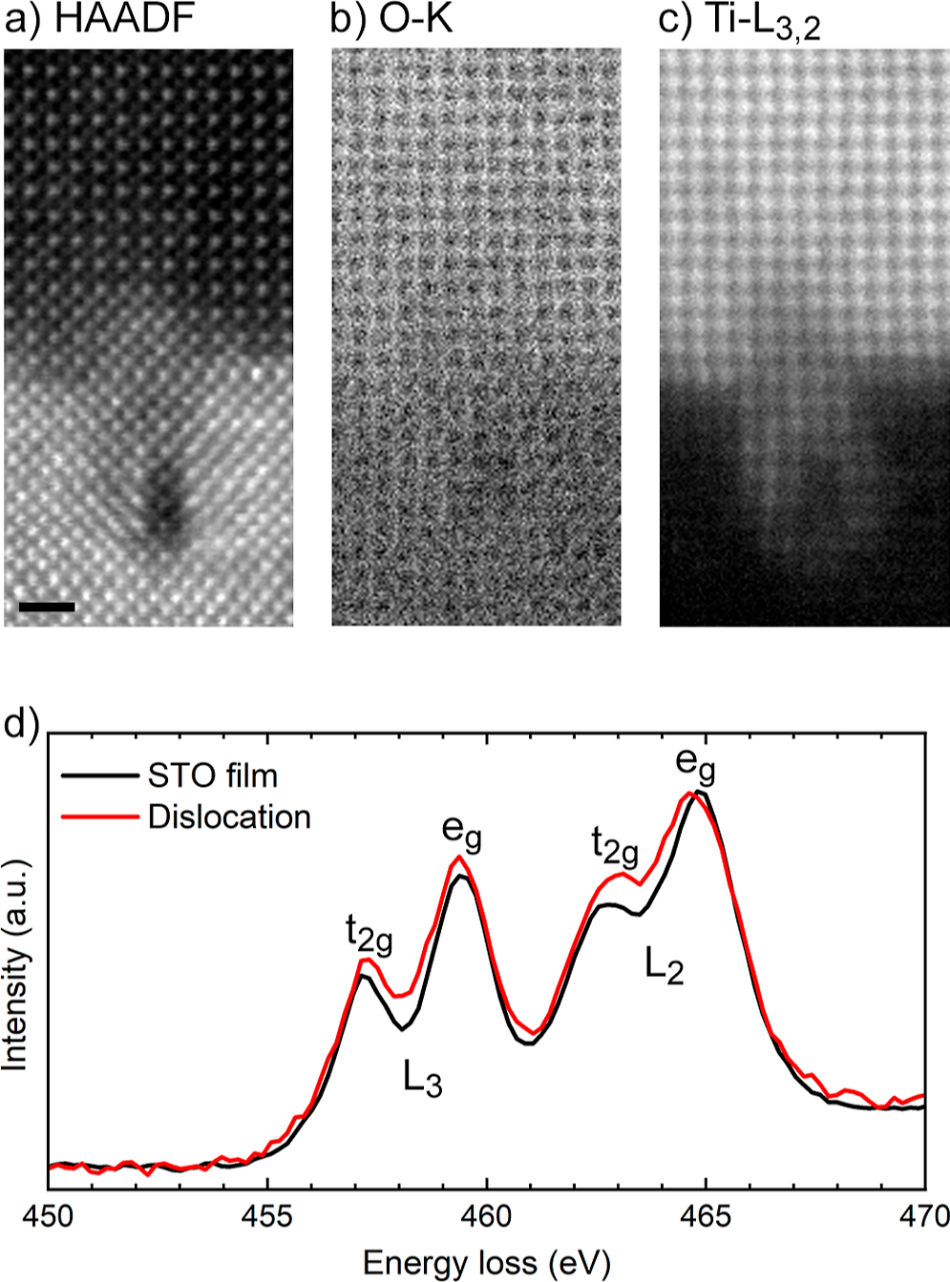
(a) HAADF-STEM image of a dislocation at the STO/LSAT interface and corresponding atomic-resolution maps of the (b) O–K and (c) Ti–L_3,2_ excitation edges. The scale bar is 1 nm. (d) Representative Ti–L_3,2_ EELS spectra of the STO film and the surroundings of the dislocation core. The spectra have been normalized to the L_2_ e_*g*_ peak height for clarity.

EELS applied to the STO/LSAT interface can be used to obtain information on both the O and Ti states from the O–K (O 1s → 2p) and Ti–L_3,2_ (Ti 2p → 3d) core edges and could thus provide information about the presence of oxygen vacancies at the interface between the two oxides. The O–K edge map of Figure 3b appears to be darker at the dislocation core, pointing to the presence of oxygen vacancies in this region. At the O–K edge, the unoccupied O p-DOS in the presence of a core hole is probed, and thus, its intensity is expected to be reduced in the presence of oxygen vacancies that are, generally, electron donors in transition metal oxides.^47^

**Figure 4:**
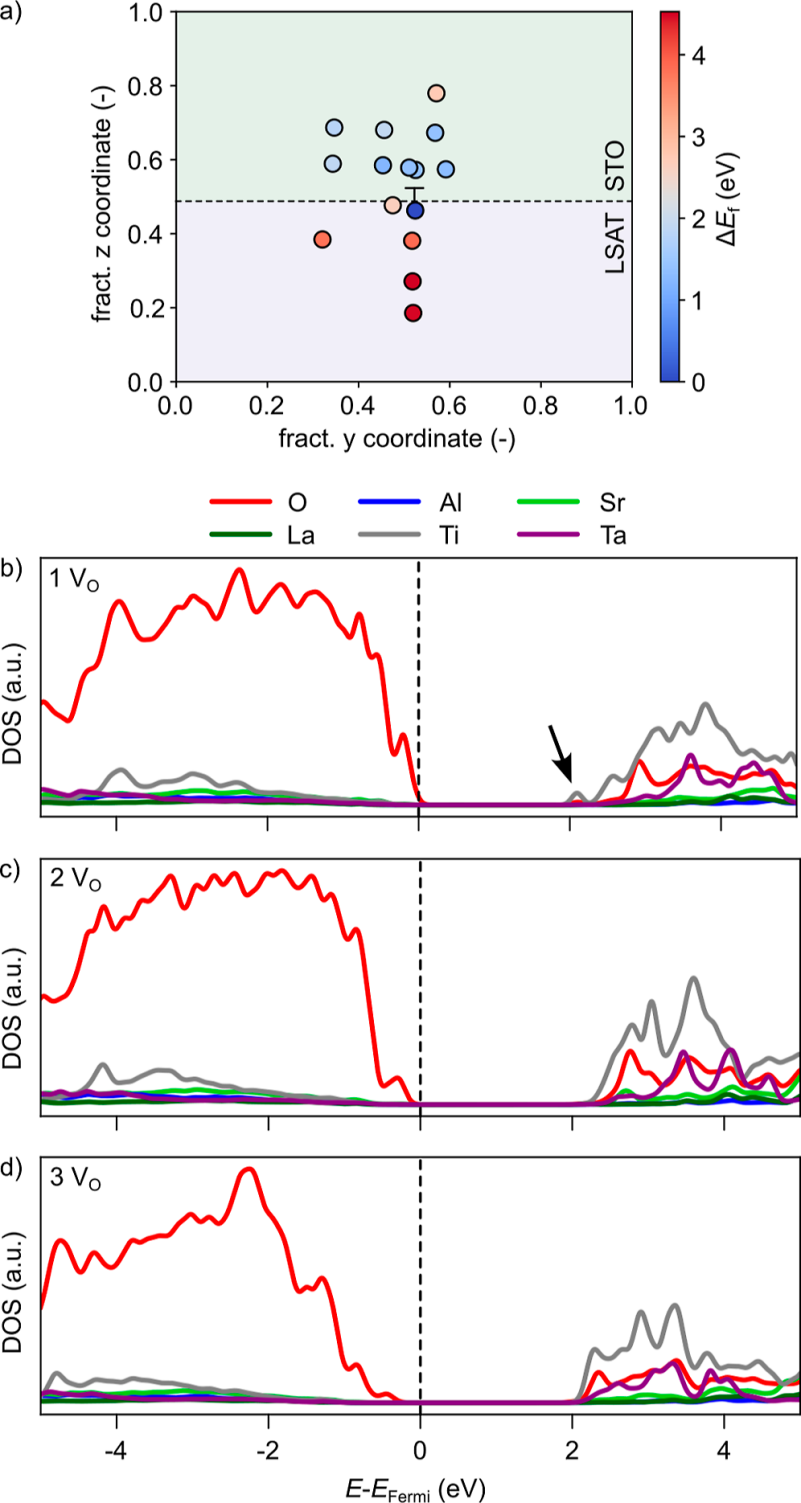
(a) Color map of the relative V_O_ formation energy (Δ*E*_f_) around the dislocation core. Electronic DOS projected on the atoms at the dislocation core for the STO/LSAT interface model containing (b) one, (c) two, and (d) three V_O_ in the most stable configurations. The vertical dashed line indicates the position of the Fermi level.

To investigate the formation of oxygen vacancies (V_O_) around the dislocation using DFT, we considered, for simplicity, a single neutral V_O_ created at different oxygen sites in our STO/LSAT dislocation model. The map reported in Figure 4a is color-coded according to the V_O_ formation energy of each V_O_ site relative to that of the most stable site (Δ*E*_f_). The energetically most favorable site to form a V_O_ is at the dislocation core for the O atoms of the extra Ta/AlO_2_ plane closest to the STO/LSAT interface. This can be ascribed to the removal of an undercoordinated O atom to form this defect. It was already observed that similar to what happens at surfaces and grain boundaries, also at the dislocation core, undercoordination can result in a lowering of *E*_f_.^20^ Furthermore, as seen above, the DOS of the stoichiometric dislocation model is characterized by hole states right above the Fermi level and localized on the undercoordinated O atoms at the dislocation core (Figure 2e). These hole states can be filled by the two extra electrons left in the lattice upon the formation of a neutral V_O_ (Figure 4b). The formation energy gradually increases with increasing distance from the dislocation core, with larger values in LSAT compared to those in STO, which is easier to reduce, as shown by the computed V_O_ formation energies in the two bulk materials (STO: 3.90 eV and LSAT: 5.04 eV). It also agrees with tensile strain, as in the STO layer, favoring neutral V_O_ formation based on chemical expansion arguments.^48^ These results suggest that V_O_ has the tendency to segregate to the dislocation core and, in particular, to the STO side of the interface, in agreement with information obtained from the O–K edge EELS spectral map.

The Ti-L_3,2_ map of Figure 3c shows that Ti atoms tend to diffuse into the LSAT. Interestingly, the Ti-L_3,2_ EELS spectra acquired in the STO film far away from the interface and around the dislocation core show striking differences (Figure 3d). Both spectra are composed of two main features, namely, the L_3_ and L_2_ edges, separated by about ∼5.5 eV due to the spin–orbit splitting of the Ti 2p core hole into 2p_3/2_ and 2p_1/2_ states. Besides, these edges are further subdivided into two peaks, the t_2*g*_ and e_*g*_ peaks, by the strong octahedral crystal-field splitting arising from the surrounding oxygen atoms. In particular, the spectrum acquired at the dislocation is characterized by a relative increase in the spectral weight of the Ti-L_3,2_ t_2*g*_ peaks, especially in the higher-energy L_2_ edge, compared with the spectrum of the STO film. Previously, such spectral changes were related to the presence of Ti^3+^ in nominally Ti^4+^-based perovskite oxides.^49^ Aside from this, the energy shift to lower energies of the Ti-L_3,2_ edge observed in the dislocation spectrum with respect to the spectra obtained in the STO film suggests the presence of reduced Ti^3+^ species in the dislocation core compared to Ti^4+^ in the STO film.^47^

This change in the oxidation state is consistent with electron doping due to neutral V_O_ at the dislocation core. Unfortunately, our DFT results did not show a complete reduction of Ti atoms at or around the dislocation core. Indeed, the DOS in Figure 4b–d do not show any filled localized state with Ti-3d character, inherent with reduction of Ti atoms to Ti^3+^ even in the presence of three V_O_, positioned, for simplicity, at the most stable sites according to Figure 4a. The absence of DOS features related to Ti reduction could be due to the limitations of our DFT method. It is well known that standard semilocal DFT functionals, such as PBE, fail in localizing the charge on Ti atoms neighboring a V_O_ for small bulk STO cells.^50^ Test calculations using a Hubbard-corrected PBE + *U* functional did not yield localized Ti^3+^ either. We note that hybrid functionals have shown promise in obtaining these excess charge locations in STO.^51−53^ Apart from being too computationally intensive for our simulation cells, these functionals may, due to the slight overestimation of the band gap, result in states that are too deep in the gap and, given the underestimation of the crystal field splitting, may have an *e*_*g*_ rather than a *t*_2*g*_ character.^54,55^ The position of the defect states is, however, improved for very large cell sizes, where oxygen vacancy defect states become shallower.^54^

**Figure 5:**
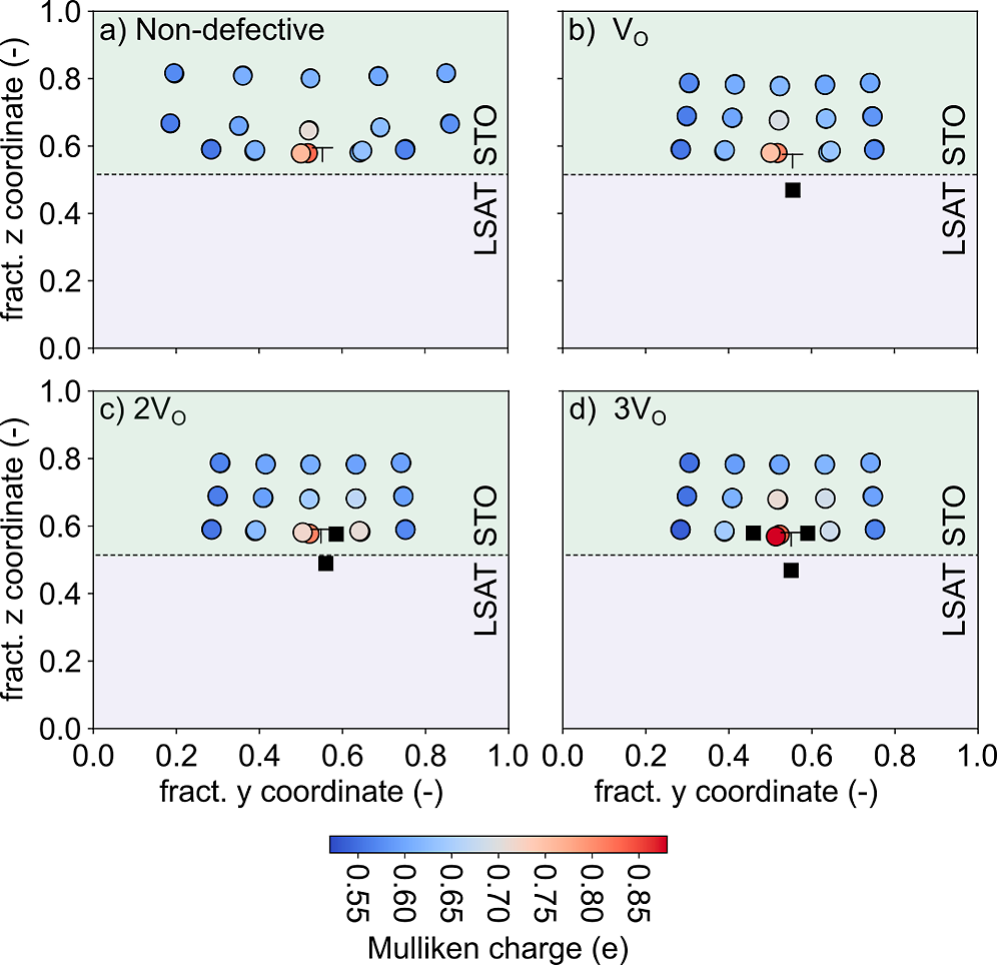
Mulliken charges for the Ti atoms in (a) the stoichiometric dislocation model and in the presence of (b) one, (c) two, and (d) three V_O_. The black squares indicate the position of the V_O_.

However, with increasing the number of V_O_, we observe a small peak with Ti-3d character at the bottom of the CB (see the arrow in Figure 4b) that is present for a single V_O_ to disappear, while the states with O-2p character at the top of the valence band are lowered in energy. This suggests a filling of Ti states for an increasing number of V_O_ and hence an increase in electron doping. This is supported by an analysis of the Mulliken charges for Ti atoms. Already in the stoichiometric dislocation model, Ti ions close to the dislocation core are more reduced compared to the rest of the Ti sites in STO (Figure 5a). The creation of the first V_O_ at the dislocation core, when the dislocation hole state is only partially filled, does not significantly alter the charge of these Ti atoms. These charges, however, increase for 2 or 3 V_O_ in STO around the dislocation (Figures 5b–d). These results suggest that the Ti^3+^ detected by EELS in the vicinity of the dislocation stems from both the presence of the dislocation and the formation of V_O_.

#### 3.2.2 Cation Vacancies

**Figure 6:**
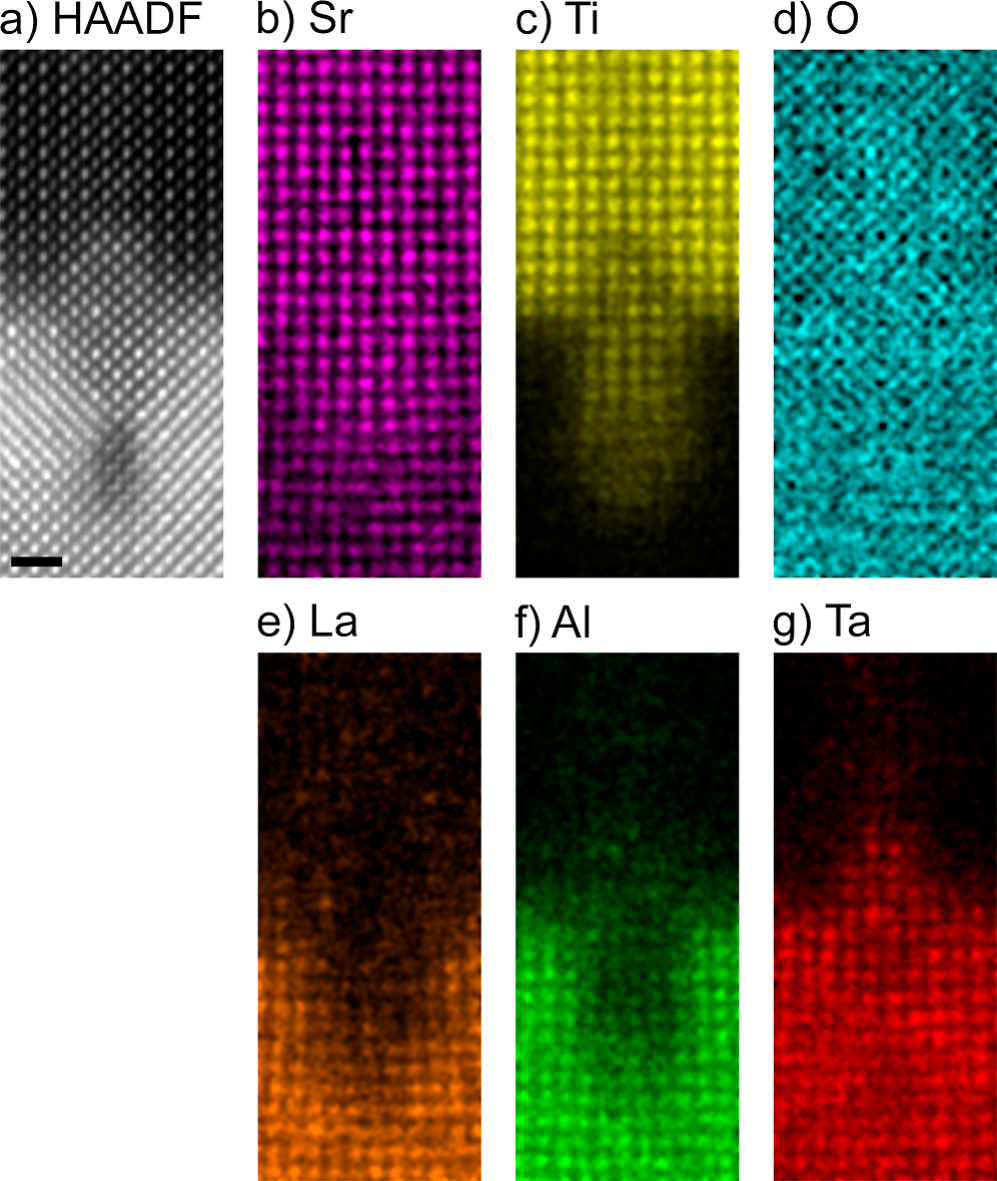
(a) HAADF-STEM image of a misfit dislocation at the STO/LSAT interface and (b–g) corresponding elemental maps of Sr, Ti, O, La, Al, and Ta calculated from an EDX spectrum image using the Sr–K, Ti–K, O–K1, La–L, Al–K1, and Ta–L lines, respectively. The scale bar is 1 nm.

To further characterize the defect chemistry of the STO/LSAT interface, we performed chemical mapping with atomic resolution using EDX. The positions of the atomic columns visualized in the EDX maps, shown in Figure 6b–g for Sr, Ti, O, La, Al, and Ta, reveal the chemical structure at the STO/LSAT interface at and around the dislocation core: cation vacancies (especially V_Al_, V_La_, and V_Ta_) are formed around the dislocation core, which are (partially) filled by Ti diffusing from STO into LSAT. At the same time, a partial substitution of Ti by Ta takes place in the STO film above the dislocation.

EDX mapping suggests that a combination of different defects accompanies dislocation formation that is driven by strain relaxation. It is, however, impractical to compute these defects simultaneously due to the size of the model and the large number of defect types and configurations that would have to be taken into account. We thus try to identify guiding rules for point defect formation in the vicinity of the dislocation core at the STO/LSAT interface by considering the most relevant defect types separately.

**Figure 7:**
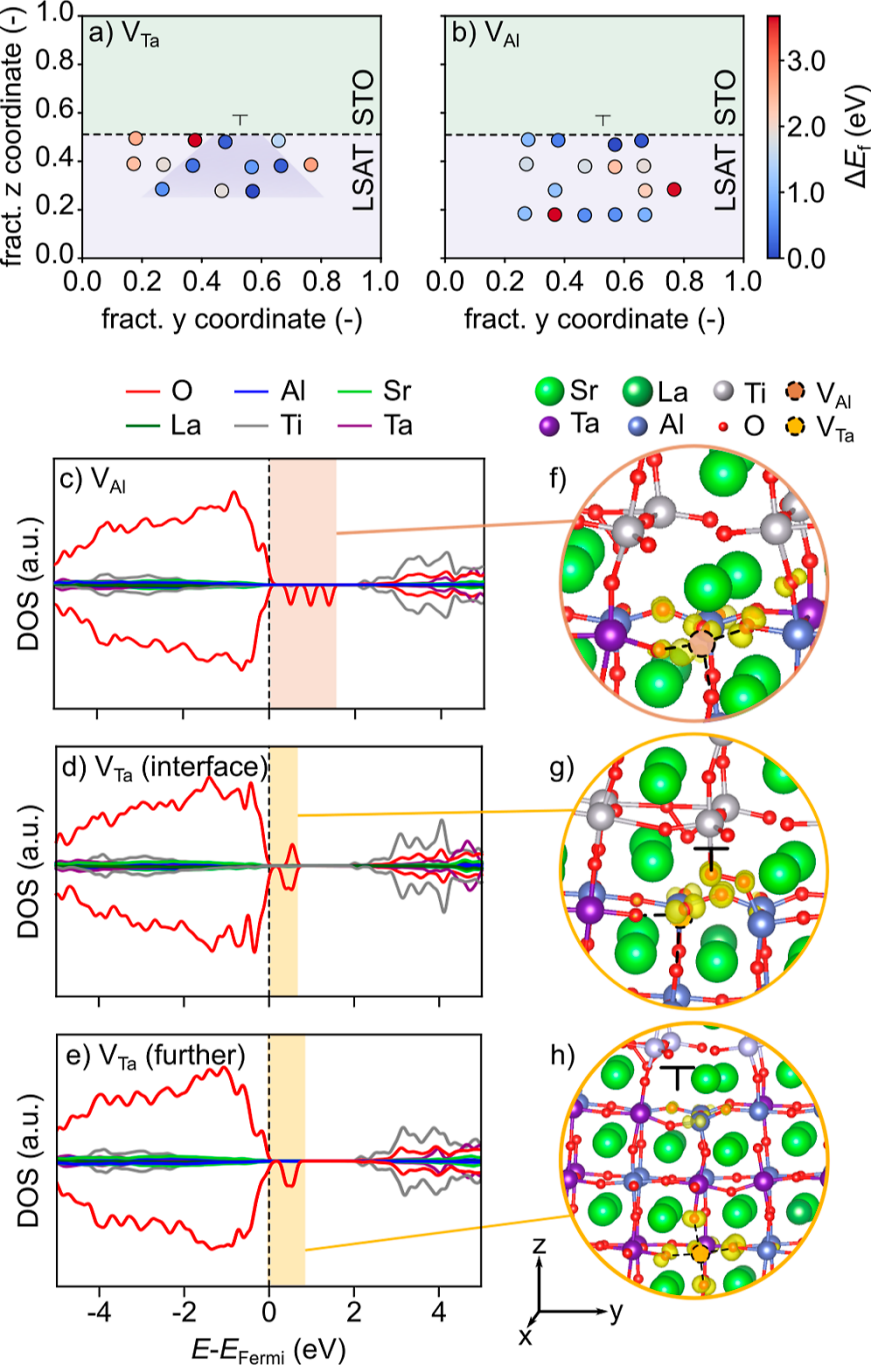
Color map of the relative (a) V_Ta_ and (b) V_Al_ formation energy (Δ*E*_f_) around the dislocation core. The energy of the most stable defect is used as a reference in each plot. The shaded trapezoidal area in (a) indicates where V_Ta_ tends to form/segregate. Electronic DOS projected on the atoms at the dislocation core containing (c) one V_Al_ and (d) one V_Ta_, each at the most stable site at the STO/LSAT interface or (e) away from the interface in LSAT. (f–h) show charge density isosurfaces (10^–2^ e/Å^3^) in the energy range of the defect states highlighted in brown or orange in the DOS.

We start by investigating the formation of a single neutral Ta (V_Ta_) and Al (V_Al_) vacancy, for which the experiment shows Ta atoms to diffuse into STO, while Al diffuses out of dislocation cores that appear elementally hollow. The map of relative formation energies in Figure 7a indicates that one of the most stable V_Ta_ configurations is at the STO/LSAT interface, closest to the dislocation core. Interestingly, all other V_Ta_ molecules have much larger formation energies (by 1.2 eV) and are thus much less favorable to form. More generally, we observe that V_Ta_ positions with low *E*_f_ values are located in a trapezoidal area below the dislocation core (see Figure 7a). This peculiar profile matches with the magnitude of the compressive strain in the LSAT region (Figure 2c), which is known to lower the formation of cation vacancies.^48^ This result suggests that V_Ta_ strongly favors a trapezoidal area below the dislocation core, in agreement with the EDX maps and the STEM image contrast (Figure 2b). Instead, V_Al_ formation is less sensitive to strain, consistent with the smaller radius of Al^3+^, favorable sites being both at the STO/LSAT interface close to the dislocation core as well as further into the LSAT substrate, in agreement with EDX mapping.

**Figure 8:**
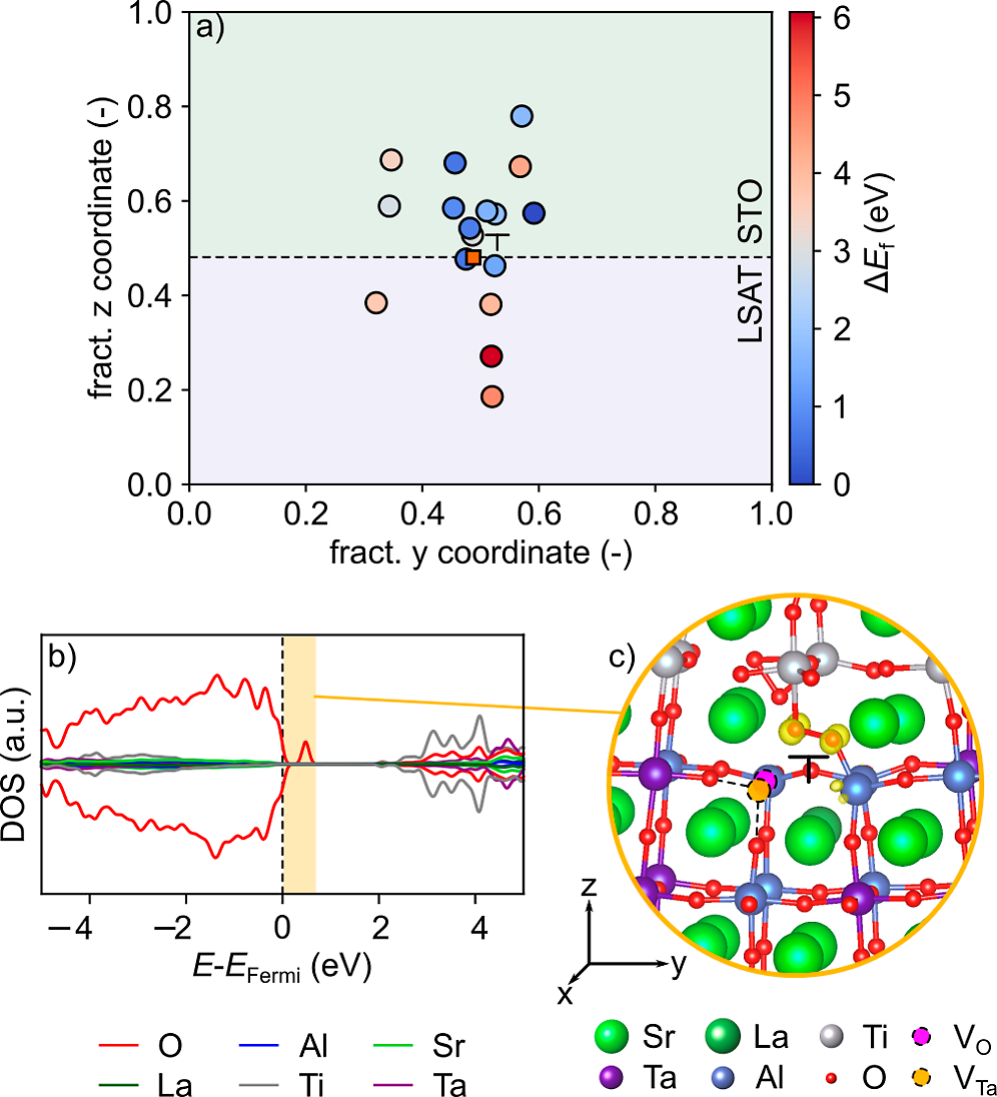
(a) Color map of the relative V_O_ formation energy (Δ*E*_f_) around the dislocation core in the presence of a V_Ta_, indicated by the orange square. (b) DOS projected on the atoms at the dislocation core containing one V_Ta_ at the dislocation core and one V_O_ at a neighboring site. (c) Charge density isosurfaces (10^–2^ e/Å^3^) in the energy range of the defect state highlighted orange in the DOS.

As expected, the formation of both V_Ta_ and V_Al_ leads to the creation of holes, as shown by the empty peaks with an O character above the Fermi level in Figures 7c–e. These states are localized on O atoms at the dislocation core when the vacancy is formed close to the core or on O atoms adjacent to the defect when the vacancy is further from the core (Figures 7f–h). Filling of these defect states at the dislocation core by electron doping, for example, due to oxygen vacancies, could further favor V_O_ formation at the dislocation core and result in conductivity along the dislocation line. For these reasons, we re-evaluated the formation of a V_O_ in the presence of a V_Ta_. The comparison of Figures 8a and 4a suggests that *E*_f_ for a V_O_ is lowered due to the V_Ta_, with a reduction of up to 3 eV for O positions close to the V_Ta_ and both in LSAT and STO. For neighboring V_Ta_ and V_O_ at the dislocation core, the excess electrons due to V_O_ formation partially heal the V_Ta_ hole state, as can be seen by comparing Figure 8b,c with Figure 7b,e.

#### 3.2.3 Substitutional Defects

**Figure 9:**
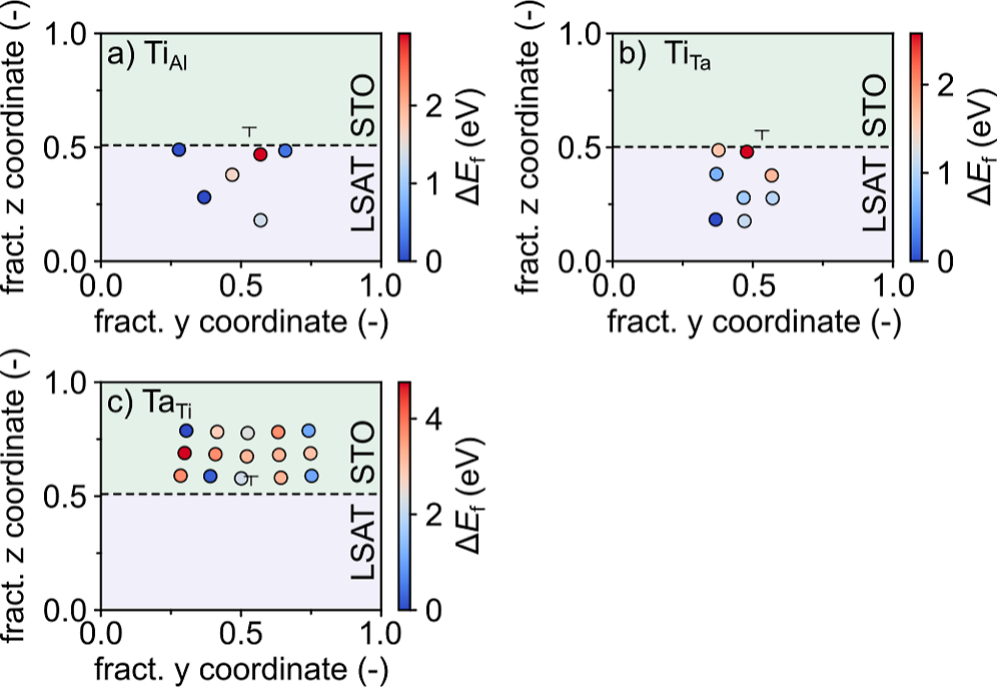
Color map of the relative (a) Ti_Al_, (b) Ti_Ta_, and (c) Ta_Ti_ formation energy (Δ*E*_f_) around the dislocation core. The energy of the most stable defect is used in each plot as a reference.

Since the interdiffusion of B-site cations, namely, Ti, Al, and Ta, between LSAT and STO is clearly visible in the EDX maps (Figure 6), we also considered the possibility of Ti substituting Al (Ti_Al_) or Ta (Ti_Ta_) in the LSAT substrate and of Ta substituting Ti (Ta_Ti_) in the STO film. When Ta_Ti_ is formed in STO (Figure 9c), we observe fairly strong variations in the formation energy for the explored configurations, with low values at the interface adjacent to the dislocation core but also further up in STO, in line with EDX maps showing Ta diffusion to 3 or even more Ti layers from the interface. Results for Ti diffusion into LSAT, forming Ti_Ta_ or Ti_Al_ (Figure 9a,b) are, instead, at odds with experiments and suggest that Ti should not be mainly located at the dislocation core, contrary to what is shown in Figure 6c.

**Figure 10:**
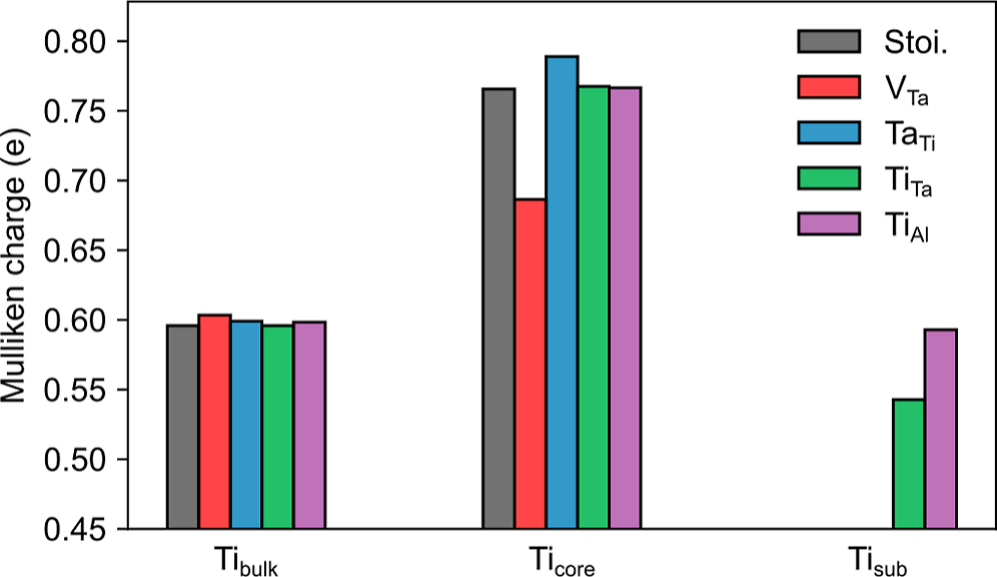
Average Mulliken charges for Ti atoms in STO far from the dislocation core (Ti_bulk_), Ti atoms close to the dislocation core (Ti_core_), and substitutional Ti atoms in LSAT (Ti_sub_).

**Figure 11:**
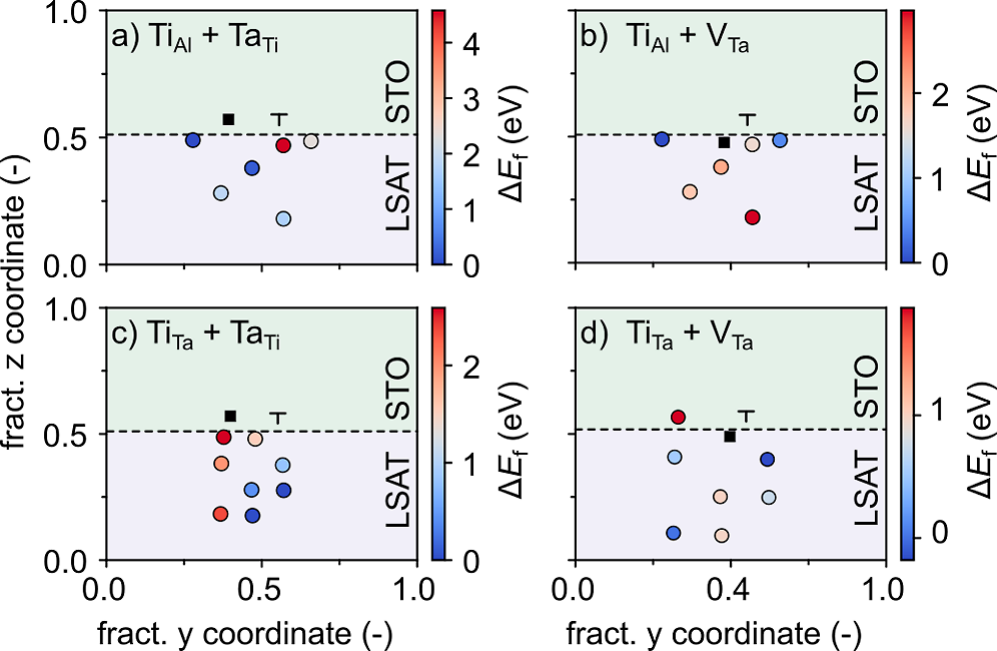
Color map of the relative formation energy (Δ*E*_f_) of a Ti_Al_ in the presence of one (a) Ta_Ti_ or (b) V_Ta_ and of a Ti_Ta_ in the presence of one (c) Ta_Ti_ or (d) V_Ta_. The energy of the most stable defect is used in each plot as a reference. The black square indicates the position of the Ta_Ti_ or V_Ta_ defect.

This result suggests that the Ti/Ta/Al intermixing observed experimentally could be the result of a complex interplay between different defect types, some of which can lead to excess charges. To further investigate the effect of electron or hole doping on the cation interdiffusion, we select the two example defects, Ta_Ti_ and V_Ta_, that result in electron and hole doping, respectively, and reevaluate Ti_Al_ and Ti_Ta_ formation in LSAT in the presence of these defects. Figure 10 reports the average Mulliken charges for Ti atoms in STO (Ti_bulk_) or at the dislocation core (Ti_core_) for different single defects in the dislocation model. This data shows, indeed, that while Ti_bulk_ stays unaltered, Ti_core_ is oxidized/reduced in the presence of a Ta_Ti_/V_Ta_ compared to the stoichiometric model. Instead, Ti-substitution in LSAT (Ti_Ta_ or Ti_Al_) does not significantly alter the charges of Ti_bulk_ or Ti_core_, but instead the substitutional Ti atom (Ti_sub_) is oxidized compared to Ti_bulk_, especially when substitution takes place at the Ta site. Comparison of Figures 9 and 11 indicates that reducing Ta_Ti_ leading to electron doping at the dislocation core favors Ti-substitution around the dislocation at less oxidizing Al^3+^ sites, while hole doping by V_Ta_ at the dislocation core increases Ti-substitution at the more oxidizing Ta^5+^ site due to charge compensation reasons. In summary, these data show that while Ti diffusion into LSAT is unlikely without other defects, it is facilitated by the simultaneous presence of V_Ta_ or by Ta diffusion into STO (Ta_Ti_).

## 4 Conclusions

In this work, we investigated the atomic structure and defect chemistry of the highly ordered Moiré network of dislocations formed at the interface between an STO thin film grown on an LSAT substrate, combining experimental and theoretical techniques. Combined MD and DFT calculations lead to an atomic-scale model of the dislocation core that features undercoordinated Ta/Al cations at the interface and has a strain field in nice agreement with the one derived from high-resolution STEM data.

Both EELS and DFT results indicate that oxygen vacancies (V_O_) easily form at the dislocation core and in the STO above the dislocation core. Furthermore, DFT calculations show that the experimentally observed Ti^3+^ around the dislocation core is due to both the dislocation structure itself and the presence of V_O_.

EDX mapping suggests that cation vacancies form at the dislocation core in LSAT: Ti substitutes Al and Ta around the dislocation core in LSAT, and a partial substitution of Ta by Ti takes place in the STO film above the dislocation core. DFT calculations confirm that cation vacancies are favored to form in a compressively strained region below the dislocation core, leading to hole doping that, in turn, further favors V_O_ formation at the core. Finally, we show that Ti diffusion into the LSAT substrate below the dislocation core only occurs in the presence of cation vacancies (favoring substitutions at Ta sites) or concurrently with the diffusion of Ta into STO (favoring substitution at the Al site).

Even though additional defect combinations and sites further from the dislocation core could be explored, the present DFT results and their good agreement with the experiment lead to a deeper understanding of the structure and electronic properties of these systems. Our results show, in particular, the predominance of p-type 1D conductivity along the dislocations, depending on the defects present also with shallow acceptor states. These results will be instrumental in further engineering the functional properties of these systems.
